# Behavioural function and development of body-to-limb proportions and active movement ranges in three stick insect species

**DOI:** 10.1007/s00359-022-01564-z

**Published:** 2022-08-20

**Authors:** Volker Dürr, Ago Mesanovic

**Affiliations:** 1 Department of Biological Cybernetics, Faculty of Biology, Bielefeld University, 33615 Bielefeld, Germany; 2 Center for Cognitive Interaction Technology, Bielefeld University, 33615 Bielefeld, Germany

**Keywords:** Body proportions, Leg length, Movement range, Allometry, Morphospace

## Abstract

Overall body proportions and relative limb length are highly characteristic for most insect taxa. In case of the legs, limb length has mostly been discussed with regard to parameters of locomotor performance and, in particular cases, as an adaptation to environmental factors or to the mating system. Here, we compare three species of stick and leaf insects (Phasmatodea) that differ strongly in the length ratio between antennae and walking legs, with the antennae of *Medauroidea extradentata* being much shorter than its legs, nearly equal length of antennae and legs in *Carausius morosus*, and considerably longer antennae than front legs in *Aretaon asperrimus*. We show that that relative limb length is directly related to the near-range exploration effort, with complementary function of the antennae and front legs irrespective of their length ratio. Assuming that these inter-species differences hold for both sexes and all developmental stages, we further explore how relative limb length differs between sexes and how it changes throughout postembryonic development. We show that the pattern of limb-to-body proportions is species-characteristic despite sexual dimorphism, and find that the change in sexual dimorphism is strongest during the last two moults. Finally, we show that antennal growth rate is consistently higher than that of front legs, but differs categorically between the species investigated. Whereas antennal growth rate is constant in *Carausius*, the antennae grow exponentially in *Medauroidea* and with a sudden boost during the last moult in *Aretaon*.

**Supplementary Information:**

The online version contains supplementary material available at 10.1007/s00359-022-01564-z.

## Introduction

In insects, adult body size and the size ratios between the main body and any of its appendages (body:limb proportions) are highly characteristic for a particular taxon. In search of an explanation for these systematic differences, different scaling rules have been proposed to govern body:limb proportions in insects, attributing their variation to biomechanical (e.g., Prange 1977; Pontzer 2007), functional (e.g. Kaspari and Weiser 1999; Weiser and Kaspari 2006; Teuscher et al. 2009; Sommer and Wehner 2012) and biogeographic (Shelomi and Zeuss 2017) reasons. However, since none of these scaling rules hold for all taxa investigated, detailed morphometric comparisons should be linked to behavioural or physiological investigations. To date, this has been done mainly for thermophilic species of ants (Sommer and Wehner 2012; Tross et al. 2021) and darkling beetles (e.g., Broza et al. 1983) with a focus on walking legs. Within the order of stick and leaf insects (Phasmatodea) body:limb proportions vary considerably between genera (e.g., Theunissen et al. 2015; Shelomi and Zeuss 2017), with conspicuous differences in the working ranges of antennae and front legs. Since these differences occur despite the same feeding biology (herbivory) and life style (nocturnal), and very similar habitats (herbaceaous vegetation and foliage of bushes and trees), it is possible that different body:limb proportions reflect different behavioural strategies of limb usage.

Additionally to the strong inter-specific variation of body:limb proportions, stick insects also show considerable intra-specific variation both among developmental stages and between sexes. For example, adult male stick insects are generally smaller and more slender than their female conspecifics, also bearing relatively longer legs and antennae. Intriguingly, strong sex-specific differences in overall body size and shape are not apparent in young larvae. Indeed, it is often tricky to tell the sex of first instar stick insects even based on characteristics of the developing external genital apparatus, let alone based on size or proportions. This raises a number of questions about the function of limbs in insects in general, and in the Phasmatodea in particular. Among these, the present study addresses the following two objectives: (i) the context-specific changes in the spatial action ranges of limbs in locomotion and active exploration, and (ii) the onset of sex-specific differences in body:limb proportions during development. For both of these objectives, we compare the species *Carausius morosus*, *Medauroidea extradentata* and *Aretaon asperrimus*. The three species represent distinct major clades of the Old World Phasmatodea (Oriophasmata: Simon et al. 2019) and differ considerably with regard to the length ratio between front legs and antennae, ranging from 1:1.4 in male *A. asperrimus* to 1:0.2 in female *M. extradentata* (Theunissen et al. 2015; their Table 1). Accordingly, they span a substantial range of limb:body proportions found in the Phasmatodea.

**Table 1:** Sample sizes of morphometric data   Insert a blank column between Stage 6 and NM in the lower part, so as to align the stage columns of males and females. See annotated proof pdf and comment there!

Females	Stage 1	Stage 2	Stage 3	Stage 4	Stage 5	Stage 6	Stage 7	*N*_F_
*Carausius*	16	12	14	12	12	12	12	90
*Medauroidea*	16	17	15	14	11	7	10	90
*Aretaon*	11	8	6	8	6	7	7	53

Males	Stage 1	Stage 2	Stage 3	Stage 4	Stage 5	Stage 6		*N*_M_
*Carausius*	–	–	–	–	7	18		25
*Medauroidea*	12	7	9	5	3	10		46
*Aretaon*	10	9	7	7	7	7		47

Number of specimens per species, stage and sex. Each specimen contributed a parameter vector comprising 58 linear body measures that were then used for all allometric and morphometric analyses. *N*_F_ and *N*_M_ give the sample sizes of females and males per species, respectively. Sample sizes per species, *N*_S_ = *N*_F_ + *N*_M_, were 115 for *Carausius*, 136 for *Medauroidea*, and 100 for *Aretaon*

With regard to inter-specific variation, we ask how differences in body:limb proportion are reflected in different usage of limbs in behaviour. It is clear that insect limbs are involved in very different kinds of motor behaviours and are key to the substantial behavioural flexibility of any species (Dürr et al. 2018). Whereas Theunissen et al. (2015) have already shown that particularly long legs come with characteristic differences in step length distribution, swing height and inter-joint coordination during stepping, the present study focuses on inter-specific differences in action range in different behaviours, relating locomotion (unrestrained, horizontal walking) to active exploration (searching) and comparing primary locomotor appendages (front legs) with dedicated sensory appendages (antennae). Among the three leg pairs we will focus on the front legs because it is clear that they are less strongly coupled to the overall step pattern than middle and hind legs (Dürr 2005; Grabowska et al. 2012), they have particular function in the initiation of turning (Dürr and Ebeling 2005) and climbing (Schütz and Dürr 2011; Theunissen et al. 2014), and they share a substantial fraction of their action volume with that of the antennae (Dürr and Schilling 2018), all of which render front legs particularly important for motor flexibility (Dürr et al. 2018).

With regard to intra-specific variation, we ask how body:limb proportions change throughout larval development, with particular focus on when and how sex-specific differences become apparent. Given that male sick insects tend to have one larval stage less than their female conspecifics (e.g., Pantel and Sinéty 1919), it is possible that differential growth of body and limbs can be related to one particular moult. For example, this could happen if growth per moult was constant in case of the thorax but not in legs, e.g., with substantially increased growth during the imaginal moult. Alternatively, sex-specific differences in body–limb proportions could arise if growth rates of thorax and legs were both constant but larger for the legs than for the thorax. To distinguish between these two alternatives, we combine classical allometry analysis (Huxley 1932) for legs and antennae with a multivariate morphospace approach (e.g., Weiser and Kaspari 2006), including 45 linear body measures to describe the overall body shape. The analysis of sex-related variation will focus on the two bisexually reproducing colonies of *M. extradentata* and *A. asperrimus*, because all laboratory colonies of *C. morosus* reproduce parthenogenetically, and the collection of males is limited by the very low likelihood of their occurrence.

## Materials and methods

### Animals

We compared three species of stick and leaf insects (Phasmatodea). Following the recent systematic analysis by Simon et al. (2019), they represent three major clades of the Old World Phasmatodea (Oriophasmata). *Carausius morosus* (De Sinéty, 1901) belongs to the subfamily Lonchodinae within the clade Lonchodinae-Necrosciinae, *Medauroidea extradentata* (Brunner von Wattenwyl, 1907) belongs to the subfamily Clitumninae, and *Aretaon asperrimus* (Redtenbacher, 1906) belongs to the family Heteropterygidae. According to Simon et al. (2019), both the Clitumninae and Heteropterygidae form distinct clades within the Oriophasmata. All three species are native to south-east Asia. *C. morosus* has been introduced to the European island of Madeira at least thirty years ago, where it has established a wild population since (Aguiar et al. 2014). Bisexual (*M. extradentata*, *A. asperrimus*) or predominantly unisexual colonies (*C. morosus*) of all three species have been bred at the Department for Biological Cybernetics at Bielefeld University for more than two decades, with recent additions of wild-caught *C. morosus* from Madeira in 2018. Note that *M. extradentata* may occur in bisexual and unisexual colonies. All colonies were kept in the same room at 23.9 ± 1.3 °C (mean ± s.d.) and a 12:12 h light dark cycle. Throughout this study, species will be addressed by their genus name.

### Behavioural experiments

Behavioural experiments were conducted on adult specimens of each species. Only one sex was studied quantitatively. The compound eyes of all animals were covered with black paint, effectively blindfolding them (none of the species has ocelli in either sex). During motion capture recordings, animals walked freely along a wooden walkway of 40 mm (*Carausius*, *Aretaon*) or 80 mm width (*Medauroidea*). Once they reached the end of the walkway, they engaged in rhythmic searching behaviour. Movements of the prothorax, head, front legs and antennae were recorded with a commercial motion capture system (Vicon MX10 with eight T10 cameras; Nexus 1.8.5; Vicon, Oxford, UK) that tracked the 3D positions of 10 light-weight, retro-reflective markers (mass: 4 mg; diameter: 1.5 mm) at a sampling rate of 200 fps and a spatial resolution of approximately 0.1 mm. Markers were attached to the body by a droplet of nail varnish. Three markers were attached to the prothorax, thus defining a body-fixed coordinate system. Further markers were placed on the head, distal femora and tibiae of both front legs, and on the first third of the antennal flagellum. An exception was *Medauroidea*, where markers were placed approximately in the middle of the flagellum, so as to maintain a minimal distance from the antennal joints to improve accuracy of angular estimates. Segment sizes and marker positions relative to the joints were measured from photographs taken under a stereolens (Olympus SZ61 equipped with Pixellink PL-B681CU). The resulting body model and the time series of all marker positions were then post-processed in Matlab (version 2019a, TheMathWorks, Natick/MA, USA) as described by Theunissen and Dürr (2013), resulting in body-centred trajectories of both antennal tips and of the tibia-tarsus joints of both front legs.

Since the transition from walking to searching does not occur simultaneously for both front legs, the onset of searching was defined separately for either body side as the instant at which the front leg tarsus lifted off the walkway to swing beyond the walkway edge. The end of searching was determined as the instant at which either the foot stepped back onto the walkway or the leg stopped moving. In *Carausius* and *Medauroidea*, these onset and stop times were the same for the ipsilateral antenna. In *Aretaon*, antennal searching often continued for a long time after the ipsilateral foot had stepped back onto the walkway. As a consequence, separate stop times were used for front legs and antennae of this species. Since the overall behaviour was very similar among specimens of one species, trials of only two or three specimens were recorded. Sample sizes were 44 trials from three female *Carausius* (*n*_1_ = 11, *n*_2_ = 14, *n*_3_ = 19), 20 trials from two male *Medauroidea* (*n*_1_ = 10, *n*_2_ = 10) and 29 trials from three male *Aretaon* (*n*_1_ = 10, *n*_2_ = 9, *n*_3_ = 10). Average trial durations were 13.6 s (range: 4.8–29.1 s) for *Carausius*, 36.4 s (range: 18.8–79.7 s) for *Medauroidea,* and 20.8 s (range: 9.6–70.0 s) for *Aretaon.*

### Morphometry

Sex and developmental stage of animals was initially judged based on the body length (head to abdomen) and, in older stages, on overall body shape and the external genital apparatus. In females, the assignment to a particular nymphal stage was confirmed based on features of the external genital apparatus of the ventral 8th abdominal segment. Male nymphal stages were generally much more difficult to assign based on features of their external genital apparatus of the ventral 9th abdominal segment. In the end, we always assigned six stages based on body size. Note that the assignment of nymphal stages was only relevant for comparison of growth curves. All quantitative morphometric analyses were independent of stage. Sample sizes used for the morphometric analysis are given in Table 1.

In case of *Carausius*, seven female stages were clearly distinguishable as described by Leuzinger et al. (1926), i.e., 6 nymphal stages and the imago. In this species, body length ranges of subsequent stages hardly overlapped, if at all. Since *Carausius* reproduces parthenogenetically and males only occur depending on environmental factors (Pijnacker and Ferwerda 1980), the number of males in the colony was very low. As a consequence, the likelihood of detecting young males is tiny, and we could collect multiple specimens of two stages, only. According to Pantel and Sinéty (1919), male *Carausius* have five nymphal stages. Lacking obvious external characteristics, we assigned animals to stages 5 or 6 (imago) based on their body length.

In case of *Medauroidea*, body length ranges of subsequent female stages overlapped considerably, particularly if Carlsberg’s key to stages was applied (Carlberg 1986; there referring to *Baculum* sp. 1). Instead, we assigned six nymphal stages (one less than Carlberg), greatly reducing the size overlap between stages while allowing for greater variation of ovipositor valve length in stages 4 and 5.

In case of *Aretaon*, we are not aware of any published description of postembryonic development and/or stage characteristics. Therefore, we monitored the development of two males and two females so as to relate increases in body length to corresponding changes in morphological features of the external genital apparatus. The resulting set of characteristics was then applied to assign six female nymphal stages (plus the imago), resulting in very little overlap of body length range between subsequent stages. As in the other species, *Aretaon* males were assigned based on body length.

Animals were sacrificed in vapour of ethyl acetate and stored in 70% ethanol. Prior to measurements, all six legs were removed at the thorax-coxa joint and all body parts were photographed with a calibrated stereolens camera (Olympus SZ61 equipped with a Pixellink PL-B681CU or Olympus DC30 digital camera). Depending on overall size and maximum limb length, 4–26 photographs were taken per specimen, following one of four sampling formats. Each sampling format specified the exact number and content of the fotos to be stored and labelled. Antennae, head, thorax and abdomen were photographed in dorsal view, with an additional ventral view of the four terminal abdominal segments for later confirmation of the sex and developmental stage. Cut-off legs were photographed from a lateral view.

**Fig. 1:**
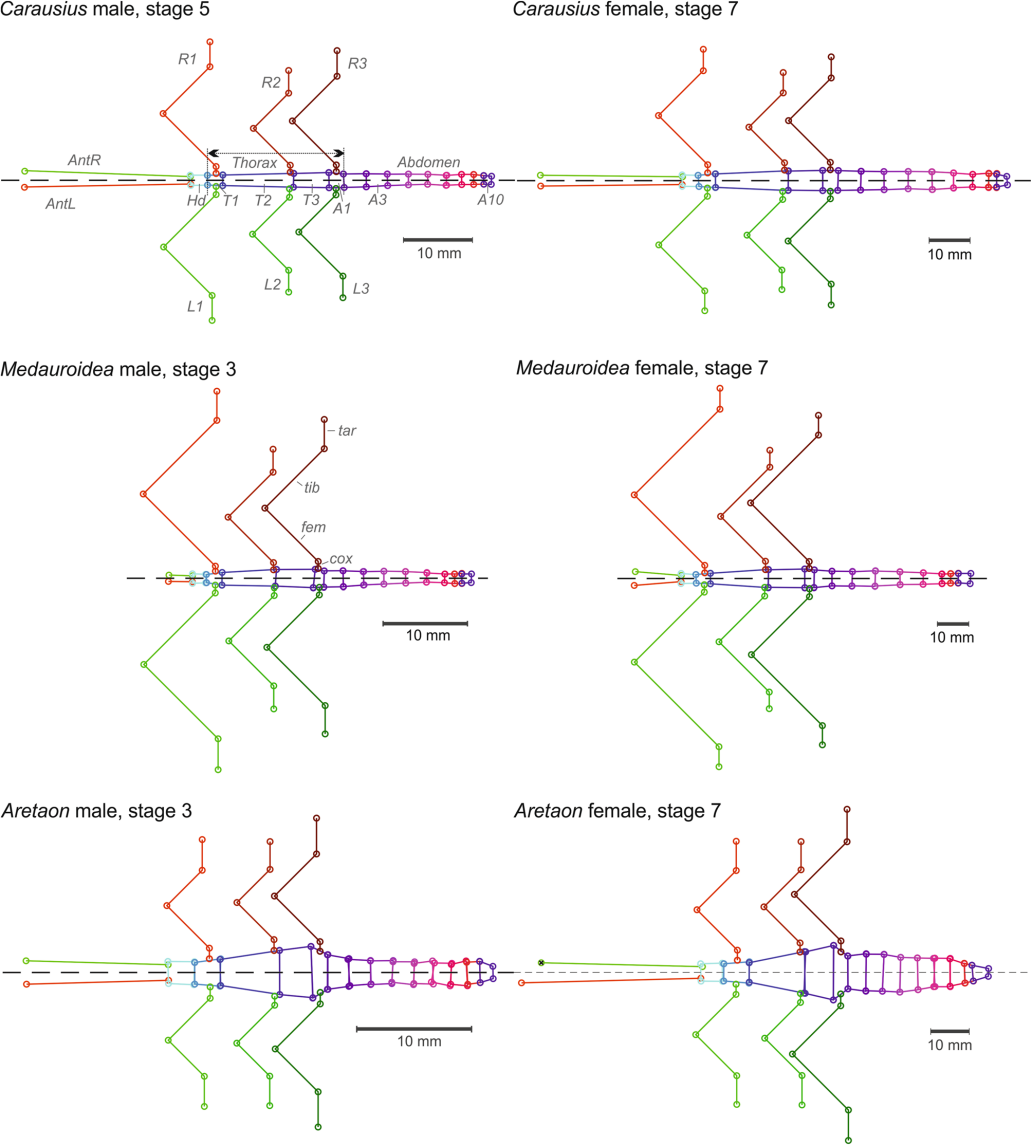
Morphometric data. Species, sex, and stage comparisons of body:limb proportions and overall body shape were based on 58 measures per specimen, as calculated from 98 image points. The six panels show representative examples of the generic graphs produced for each specimen. Rows show two specimens per species, the left being a male of the 3rd or 5th nymphal stage, and the right being an adult female (stage 7). Labels of body segments and limbs as used throughout this study are given for the male *Carausius* (top left: Hd: head; T1 to T3: thorax segments; A1–A10: abdominal segments; L1–L3: left legs; R1–R2: right legs; AntL/AntR: left and right antenna). Note that A1 is fused to T3 in Phasmatodea. As the hind legs attach below the tergite of A1, the leg-bearing tagmon “Thorax” is generally considered to include A1 (see arrow). The four segments of the walking legs are labelled for the right hind leg (R3) of the male *Medauroidea* (middle left). Whenever a labelled body part was considered as “not intact” this was indicated by a black cross, as is the case for the right antenna (AntR) of the female *Aretaon* (lower right). Points from non-intact body parts were excluded from the analysis

A custom-written “BodySizeLogger App” was programmed in Matlab. This program allowed automated, sequential access to all images belonging to the same specimen, and instructed the user which body feature to mark next. Accordingly, users manually labelled the same set of body features with mouse clicks, and tagged whether or not the labelled body part was intact. For example, the antennae and tarsi sometimes lacked the distal part, and these “defect” body parts would be excluded from further analysis. If necessary, images could be zoomed-in to improve labelling accuracy. A minimum of 96 points were labelled per image set (at least 2 per antenna, 4 per head, 18 per thorax, 40 per abdomen, and 5 per leg) and stored in one file per specimen. Figure 1 shows schematic body shapes of six specimens based on the manually labelled body features.

Lengths of body segments were measured from the positions of the lateral segment borders. The only exception was the 1st abdominal segment (*A1*) which, in the Phasmatodea, is fused with the metathorax (*T3*) and often has a curved anterior border. The position of T3-A1 border was set to its dorsal midline position. Segment widths were calculated as the means of the anterior and posterior segment widths, thus neglecting the tapering shape of some segments (e.g., of T3). In total, 58 linear body measures were used to describe the overall body shape and size, comprising 30 measures of limb positions and segment lengths (the lengths of both antennae, *Ant*; 3 × 4 leg segments per body side, i.e. coxa, *cox*, trochanterofemur, *fem*, tibia, *tib*, and tarsus, *tar;* the lateral distance of the head-scape joints relative to the midline and the 3 distances of the thorax-coxa-joints relative to their posterior segment boundaries), and 28 measures describing the shape of the main body (lengths and widths of the head, *Hd*, thoracic segments *T1*–*T3*, and abdominal segments *A1*–*A10*).

Throughout this paper, *body length* was calculated as the sum of all body segment lengths (*Hd, T1*–*T3,* and *A1*–*A10*), *thorax length* was calculated as the sum of the three thorax segment lengths (*T1*–*T3*) plus the length of *A1*, and *leg length* was calculated as the sum of the measures *cox* + *fem* + *tib* + *tar*.

### Data analysis

All data processing and statistical calculations were done in Matlab (version 2019a, TheMathWorks, Natick/MA, USA). Working ranges of antennae and front legs were analysed for pooled data from each species, and normalised with respect to the length of the front leg. Ranges were visualised for the entire set of trajectory points per limb or by the convex polygon surface surrounding the corresponding point density clouds on a 1.33 mm grid in *Carausius* and *Aretaon*, and a 2.17 mm grid in *Medauroidea* (approximately 3% of the longest limb length). To obtain reasonably smooth estimates of the working range boundaries, point density clouds were smoothed by a 3D Gaussian kernel (size: 5 × 5 × 5 voxels). Volumes included all voxels with at least 1% of the maximum point density. Point density clouds, thresholds and volumes were calculated separately for each limb, thus accounting for asymmetries of limb length, marker placement and/or accuracy. For example, antennal markers were not placed at equal distances, resulting in a left–right asymmetry of antennal elevation measures and improved tip accuracy for the antenna with the more distant marker. For further computational details see Dürr and Schilling (2018).

Based on file lists of individual BodySizeLogger files, summary tables were generated for overview and visual inspection. All computations were done in Matlab. 95% confidence intervals per stage were calculated as *qt*(0.975, *n*−1)·*s*/$\sqrt{n}$, where *qt* is the 97.5% quantile of the *t*-distribution with *n*−1 degrees of freedom, s is the standard deviation and n is the sample size. Allometric relationships were described by power functions or—in two cases—exponential functions and visualised as log–log graphs with limb length depending on thorax length. The corresponding scaling functions were fitted as linear regressions to log-transformed measures of limb length, *y*, and thorax length, *x*, such that the resulting slope, *b*, and intercept, log(*a*), gave parameter estimates for the linear function log(*y*-*y*_0_) = *b*·log(*x*) + log(*a*), corresponding to the power function *y–y*_0_ = *a·x*^*b*^. In case of the antennae of *Medauroidea*, linear regressions were calculated for the semi-logarithmic relationship log(*y*–*y*_0_) = *b*·*x* + log(*a*), corresponding to the exponential function *y–y*_0_ = *a·e*^*b·x*^. In both cases, coefficients of determination, *r*^2^, were calculated on the transformed data.

Principal Component Analysis (PCA) was calculated on samples of log-transformed parameter vectors that comprised 45 linear body measures. These were a subset of the 58 measures listed above, rejecting the smaller one of each bilateral pair of leg segment lengths or antenna lengths. Sex-related differences in the development of body shape were then analysed separately for each species. For this, PCA was calculated on the N_S_ parameter vectors per species (see Table 1), excluding specimens with any non-intact body features. Each parameter vector was described by a linear combination of all principal components, PC, yielding 45 scores per specimen, one for each PC. The change in body shape during development was then assessed based on the mean scores per stage of the first three PC.

## Results

### Spatial searching behaviour and working ranges

**Fig. 2:**
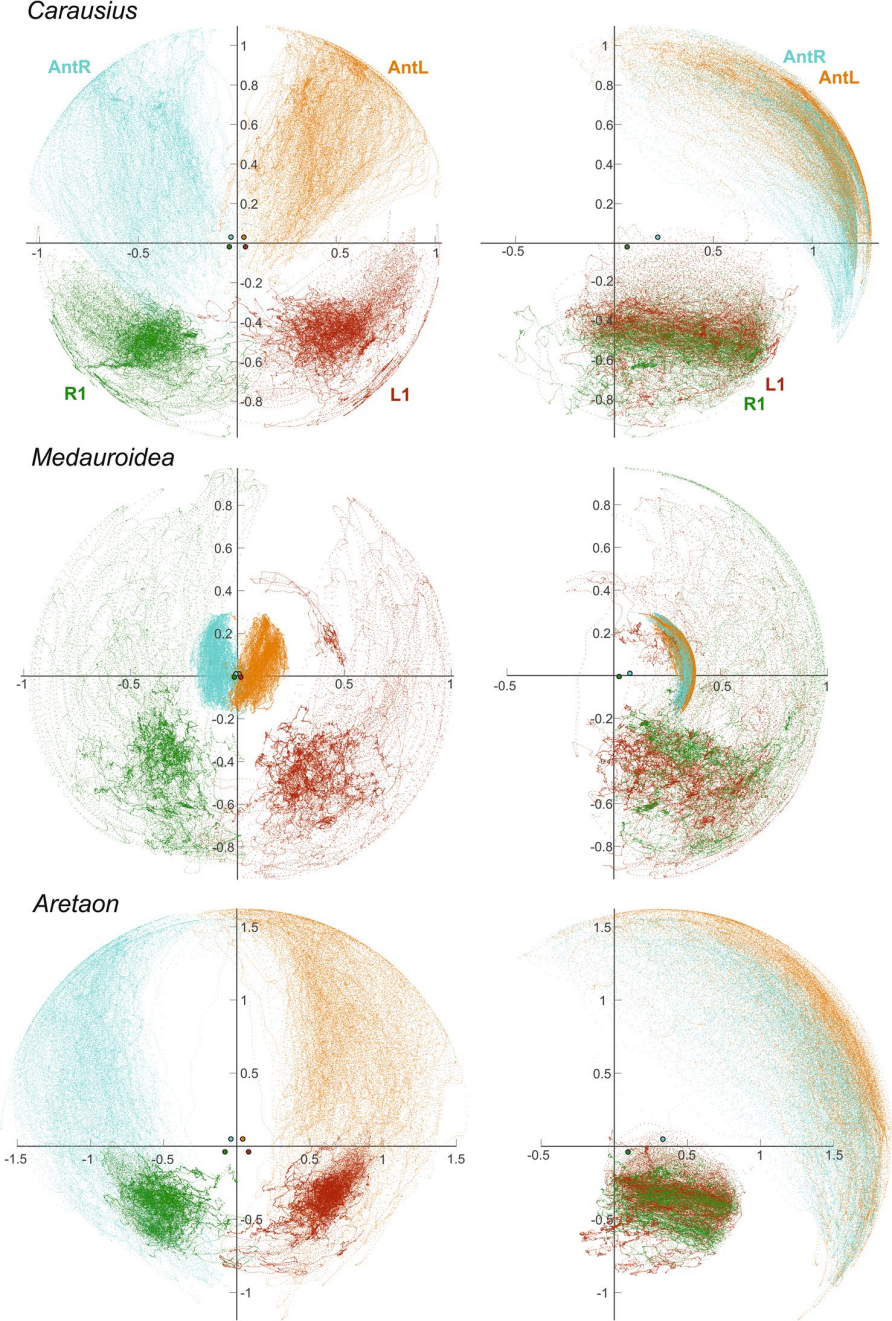
Movement ranges of front legs and antennae during walking. Panel rows correspond to different species. Each row shows a frontal (left panel) and sagittal (right panel) view of the pooled, spatial distribution of antennal tip positions (cyan: right antenna, AntR; orange: left antenna, AntL) and of the tibia endpoints of the left (L1; red) and right (R1; green) front legs. Distances were normalised to the front femur + tibia length (per animal) and positions are drawn relative to the mean locations of the head-scape joints (for AntL and AntR) and prothorax-coxa joints (for L1 and R1) that are shown as filled circles near the graph origin. Note that the number of points per species differ, but are the same for all four limbs per species. For trial numbers and mean durations of walking episodes see Table 2

Following considerations about dual function of walking legs in locomotion and near-range exploration (Dürr et al. 2018) the main objective of the behavioural analysis was on inter-species differences in the spatial movement ranges of front legs, how this related to the concurrent movement range of the antennae, and whether movement ranges changed with the transition from walking to searching. All three species rhythmically moved their antennae as they walked along the walkway (Fig. 2), irrespective of antenna length. To illustrate the relative lengths of antennae and front legs, the axes of Fig. 2 were normalised to the sum of femur + tibia lengths. The side views in Fig. 2 (right panels) show that the radius of the antennal working range (cyan and orange) was less than 0.5 for *Medauroidea*, approximately 1.2 for *Carausius*, and well beyond 1.5 for *Aretaon*. Owing to the shortness of its antennae, *Medauroidea* is unlikely to touch anything with its antenna before a front leg had reached it. Accordingly, continuous antennal movement in this species will not contribute to detection and tactile localisation of objects and must have another reason.

Apart from continuous antennal movement during walking, all species transitioned from walking to searching behaviour in the same way: as soon as a front leg stepped across the terminal edge of the walkway, it engaged in rhythmic searching movements with the foot moving along cyclic loops.

**Fig. 3:**
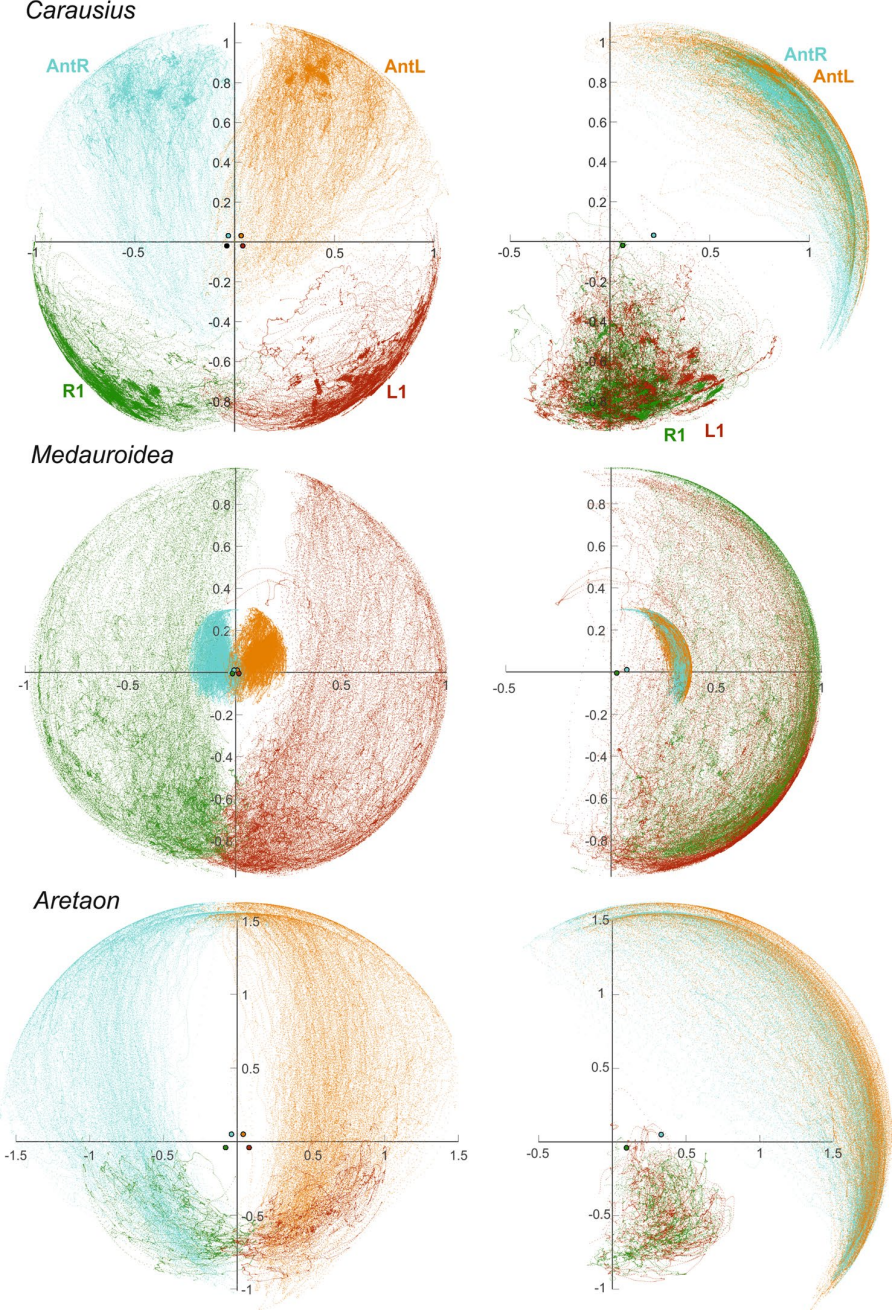
Movement ranges of front legs and antennae during searching. Data from the same trials as in Fig. 2, but for immediately subsequent searching episodes. Same graph details as in Fig. 2. Note that in case of *Aretaon* (lower panels), the point numbers for antennal tips and front legs differ because searching terminated much earlier in front legs than in antennae. For trial numbers and mean durations of searching episodes see Table 2

This transition was side-specific in that the contralateral leg maintained ground contact for a duration consistent with one step period (*Delay* in Table 2). In all animals tested, the fraction of trials in which this walk-to-search transition occurred first on the right body side was not statistically different from 50% (Exact binomial tests: *p* > 0.265; see *L/R* and *N* in Table 2). The transition from walking to searching was less conspicuous in antennal movement, as both antennae continued to be moved in a similarly rhythmic manner as during walking (Fig. 3).

**Table 2:**
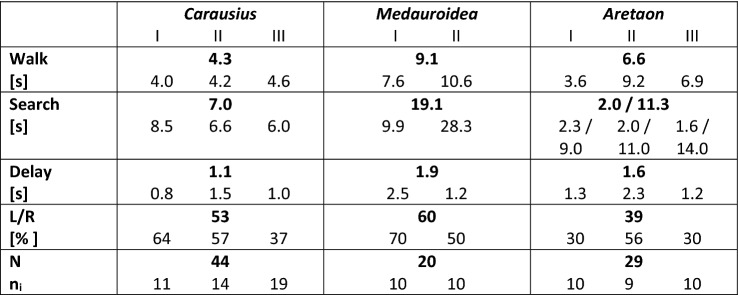
Mean walk and search durations

Mean durations of walking and searching episodes, along with the delay of searching onset between left and right legs. In case of *Aretaon*, the duration of the searching episode was determined separately for legs and antennae. The second, higher values stand for the antennae. L/R gives the percentage of trials in which the search started with the right front leg. Bold numbers are means of per-animal means. Numbers below give per-animal means, labelled with roman numbers I–III

The main differences between species concerned (i) the persistence of searching episodes, (ii) the mode of termination of searching, and (iii) the overall searching ranges of front legs and antennae. *Carausius* and *Medauroidea* typically terminated searching at the same time for front legs and antennae, either by assuming a static posture with outstretched limbs, or by resuming ground contact with the front legs and turning around on top of or climbing under the walkway. In contrast, *Aretaon* tended to terminate the searching-movement of front legs after very few loops, then resuming ground contact of the foot while the ipsilateral antenna continued searching much longer (see *Search* in Table 2 with separate values for front legs and antennae in case of *Aretaon*). Overall, searching episodes were longest in *Medauroidea*, where they regularly persisted for 20 s, and shortest for *Carausius*, where mean durations per animal ranged between 6 and 8.5 s, only.

Since the walked distance was the same in all species, differences in the durations of walking episodes (*Walking* in Table 2) corresponded to different walking speeds. Therefore, the long-legged *Medauroidea* walked most slowly. Note that the different durations listed in Table 2 are reflected by point densities in Figs. 2 and 3, as sampling intervals were the same for each species. For the same reason, local density differences of foot positions in Fig. 2 reflect the speed of swing and stance movements as well as the fact, that the foot spends a lot more time in stance (foot on substrate) than in swing (foot in air). Foot trajectories differed very strongly among species. Whereas even swing movement trajectories stayed below the body midline (lower quadrants in panels of Fig. 2) for most of the time in *Carausius* and *Aretaon*, they covered the entire frontal hemisphere in *Medauroidea*. This confirms an earlier finding that *Medauroidea* swing movements are much higher than in species with shorter relative leg length (Theunissen et al. 2015), and additionally shows that swing movements are subject to very strong spatial variation. Owing to this spatial variation, the front feet could point into virtually any direction within the frontal hemisphere. In contrast, swing trajectories appeared much less variable in the other two species, though with greater elevation in *Carausius* than in *Aretaon*.

The antennal movement ranges during walking differed in both the medio-lateral and dorso-ventral angular ranges (see front and side views of point clouds in Fig. 2, respectively). The dorso-ventral range was largest in *Aretaon*, where it spanned approximately 140° (ca. from 110° levation to − 30° depression). In comparison, the short antennae of *Medauroidea* covered only about 90° (ca. 70° levation to − 20° depression), whereas *Carausius* covered some 110° (ca 90° levation to − 20° depression). Thus, the dorso-ventral angular range correlated with the relative length of the antenna, being largest in *Aretaon*, smallest in *Medauroidea*, and intermediate in *Carausius.*

**Fig. 4:**
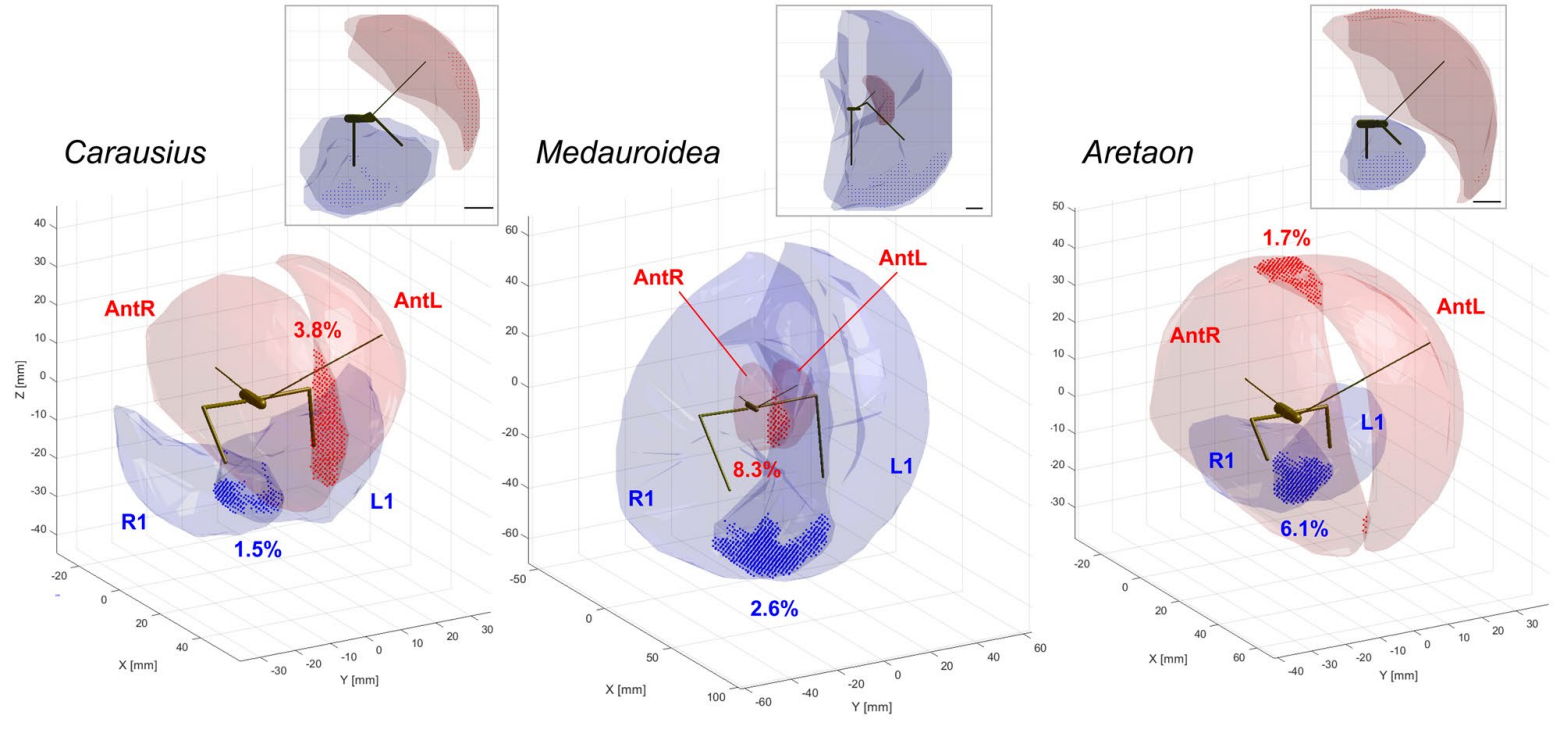
Near-range exploration volumes of antennae and legs differ in size, range and overlap. *Large panels* show near-range exploration volumes (translucent surfaces) of the two antennal tips (light red; AntR: right antenna; AntL: left antenna) and two tibia-tarsus joints (light blue: R1: right front leg; L1: left front leg). Dotted regions show bilateral overlap ranges of antennae (red) and front legs (blue). Percentages give the fraction of the bilateral volume that was traversed by both limbs. Volumes include both walking and searching episodes, and voxels with at least 1% of maximum density. Top right inserts show the same volumes in side view, illustrating the complementary “exploration effort” of antennae and front legs. The spacing of the overlap symbols indicates the resolution of the 3D grid used for the analysis

The medio-lateral movement ranges of the two antennae overlapped in front of the head in *Carausius* and *Medauroidea*, whereas they left a fairly large medial region unsampled in *Aretaon* (see blank region between cyan and orange clouds in front view). This changed only little during searching (Fig. 3), as the left and right antennal movement ranges of *Aretaon* overlapped slightly more at the midline just below the head (see overlap zones in Fig. 4). Overall, spatial antennal movement ranges differed only little between walking (Fig. 2) and searching (Fig. 3). This was different in case of the front legs, where the lack of ground contact during the searching episode allowed lower foot positions and a more even coverage of the movement range than during walking.

In general, spatial movement ranges of front legs and antennae were complementary during searching, such that almost any spatial direction within the frontal hemisphere was covered at least up to the radius of the front leg length. To illustrate this, Fig. 4 shows the sizes, shapes and locations of the near-range exploration volumes of the antennae and front legs, along with their bilateral overlap regions. In none of the species did the near-range exploration ranges of antennal tips and tibia-tarsus joints overlap. Rather, the angular ranges of antennae and front legs were complementary in that they covered different sectors in case of *Carausius* and *Aretaon* (see side views in lower panels of Fig. 4) and different distances in case of *Medauroidea* (in Fig. 4 the blue envelopes fold over red envelopes).

The strongest difference between walking and searching episodes concerned the bilateral overlap of the near-range exploration volumes (see symbols in Fig. 4). The change was most pronounced in *Aretaon*, where the fraction of overlap increased from 0.6 to 1.7% in case of the antennae, and from 3.0 to 6.1% in case of the front legs (percentages give fractions of the total bilateral volume). For comparison, in *Carausius* the increase was from 2.3 to 3.8% in case of the antennae, and from 0.9 to 1.5% in case of the front legs. In *Medauroidea*, the bilateral overlap more than tripled in case of the legs (from 0.8 to 2.6%) whereas it slightly decreased in case of the antennae (9.7–8.3%). The overall sizes of the near-range exploration volumes changed mainly for the legs. This is consistent with the fact that the walkway imposed a spatial boundary during walking, but not during searching. Accordingly, near-range exploration volumes of the legs during walking were always smaller than the total volumes. The difference was largest in *Medauroidea* (63% of the total volume), smallest in *Carausius* (97%) and intermediate in *Aretaon* (83%).

### Limb proportions, allometry and sexual dimorphism

**Fig. 5:**
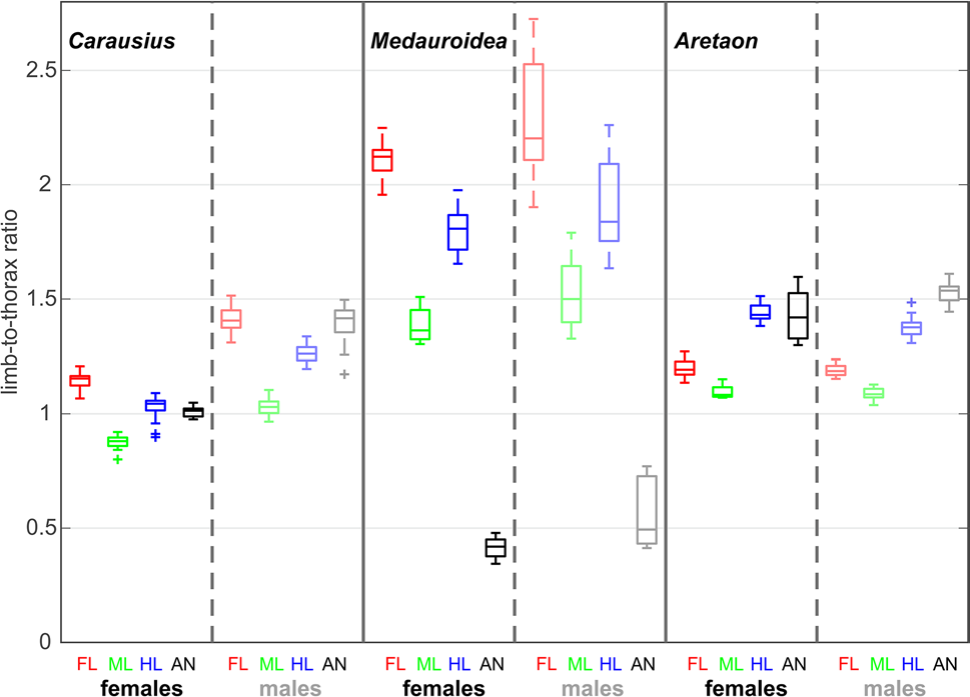
Limb-to-thorax length ratios of adult stick insects. Box plots show samples of ratios of limb length over thorax length for adult animals, with females (dark colours) and males (lighter colours) juxtaposed (Red: front legs; Green: middle legs; blue: hind legs; black: antennae). Thorax length includes the length of the 1st abdominal segment, A1. Leg lengths are sums of the four segment lengths coxa, trochantero-femur, tibia and tarsus. For sample sizes and test statistics see Table 3

Given our finding that relative limb length was mirrored by the spatial movement ranges and persistence of searching, we wanted to know how relative limb length varied with sex and developmental stage. In adult animals, relative limb length was sexually dimorphic within each one of the three species, though consistently different among species. The comparison of limb-to-thorax length ratios of adult specimens in Fig. 5 reveals a characteristic pattern for each species, albeit with statistically significant differences between the sexes for all limbs of *Carausius*, three out of four limbs in *Medauroidea* (only the hind leg has the same ratio in both sexes) and in the hind leg of *Aretaon* (Table 3). While males had larger limb-to-thorax length ratios in *Carausius* and *Medauroidea*, it were the females in case of *Aretaon*.

**Table 3:** Sex difference of limb-to-thorax length ratios

	*Carausius*		*Medauroidea*		*Aretaon*	
	*p*	*n*_1_, *n*_2_	*p*	*n*_1_, *n*_2_	*p*	*n*_1_, *n*_2_
Front leg	** < 0.0001**	24, 35	**0.0322**	19, 18	0.5542	14, 12
Middle leg	** < 0.0001**	24, 34	**0.0018**	20, 20	0.5768	13, 14
Hind leg	** < 0.0001**	22, 31	0.1062	20, 19	**0.0034**	11, 14
Antenna	** < 0.0001**	16, 23	**0.0017**	17, 17	0.0556	7, 12

*p* values and sample sizes for Wilcoxon’s ranksum test on medians of limb-to-thorax length ratios of adult male and female stick insects, as shown in Fig. 5

Bold numbers indicate significance level *p* < 0.05

In all species, the middle leg was the shortest leg. Two characteristic differences among species concerned the very long legs and very short antennae of *Medauroidea* (having the highest and lowest limb-to-thorax ratios in Fig. 5, respectively) and the hind legs and antennae being the longest limbs in *Aretaon* (in *Medauroidea* and *Carausius*, the front legs are longest). Both of these characteristics hold for either sex.

**Fig. 6:**
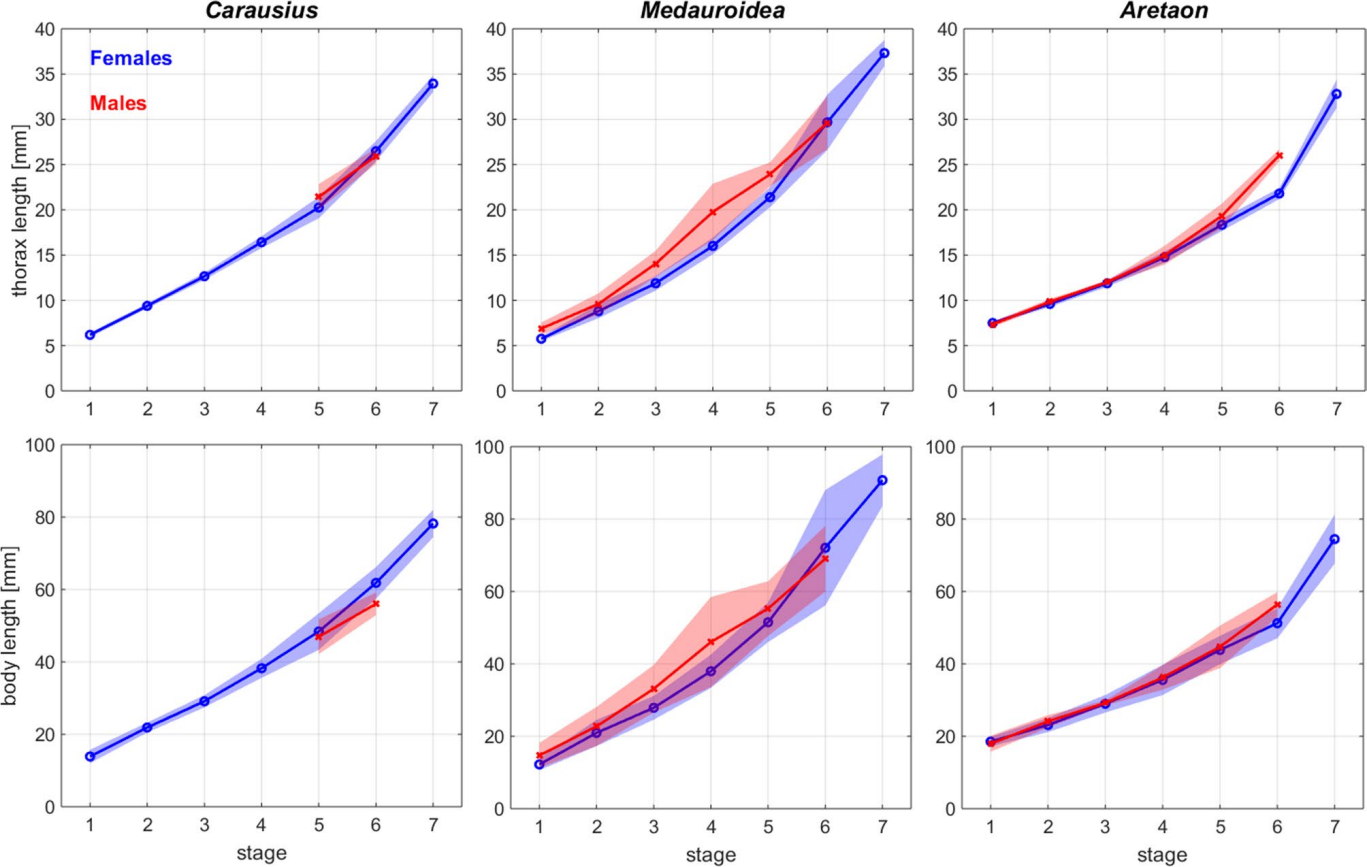
Growth curves for thorax and body length. Thorax length is less variable than body length. Top panels show mean thorax length per developmental stage for females (blue) and males (red) of the same species. Bottom panels show the corresponding growth curves for the length of the main body (from head to 10th abdominal segment). Shaded areas show 95% confidence intervals of the mean. For sample sizes see Table 1

Generally, we used thorax length to express body:limb proportions rather than “full body length” (head + thorax + abdomen). This was because thorax length proved to vary less than body length. To illustrate this, Fig. 6 shows the growth curves for both of these measures, with 95% confidence intervals per stage. Thorax length per stage proved to be distinctly different between the sexes of *Medauroidea* nymphs (stages 1–5) and in stage 6 of *Aretaon* (adult males and last nymphal stage in females), as illustrated by little or no overlap of the 95% confidence intervals. Note that stage assignment of males was based on total size and, more generally, numbers of moults may vary within the same species.

Body length measures of the exact same samples varied a lot more than thorax length measures, as illustrated by the strong overlap of 95% confidence intervals (lower panels of Fig. 6). We attribute this difference in variability to the mechanical properties of the thorax exoskeleton which is much less compliant than the abdomen. Particularly the abdomen of young nymphs is fairly soft, and lengthening of the abdomen is known to occur between moults (e.g., Ling Roth 1917), thus increasing length variation per stage. Finally, thorax length is of more immediate relevance to locomotor function because it affects the “base distance” between legs. Therefore, the limb-to-thorax length ratio seems more appropriate for interpreting limb function than the limb-to-body length ratio.

**Fig. 7:**
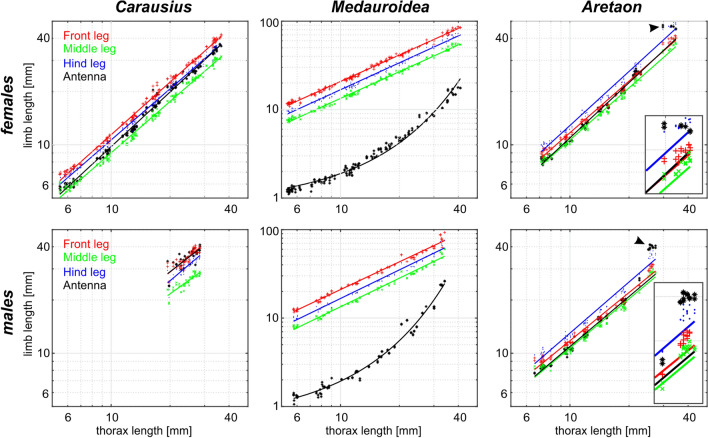
Antennae grow differently than legs. Log–log plots showing allometric growth of limb length as a function of thorax length in females (top panels) and males (bottom panels). Red: front legs; green: middle legs; blue: hind legs; black: antennae. Solid lines show linear regressions in a log–log plot or—in case of *Medauroidea* antennae—in a semi-logarithmic plot. Thus, straight lines correspond to allometric power functions and curved lines correspond to exponential functions (black curves in mid panel). Arrow heads and enlarged inserts in *Aretaon* panels indicate that antenna (black) and hind leg (blue) length of adult animals strongly deviate from the linear regression. For regression parameters see Table 4

To assess postembryonal development of limb proportions, we determined the allometric growth of all limbs with respect to the corresponding change in thorax length. Expecting power law relationships between thorax length and limb length, Fig. 7 shows double-logarithmic plots of these measures. Since logarithmic transformation linearizes power functions, the slope, *b*, and intercept, log(*a*), of the resulting linear dependencies yielded parameter estimates for the underlying power law. In case of the walking legs, Fig. 7 confirms that leg length was generally well-described by a power law with an exponent close to unity (*b* in Table 4) and linear regressions explaining more than 97% of the variance (*r*^2^ in Table 4). The only exception were *Carausius* males. Furthermore, regression slopes were similar for the three leg pairs per species (i.e., red, green and blue lines in Fig. 7 are nearly parallel in most cases), showing that limb-to-thorax proportions did not change much during postembryonic development. A notable exception were the legs of adult *Aretaon*, where the increase in length from the last nymphal stage to the imago was considerably larger than the corresponding increase in thorax length. As a consequence, most measures of adult leg length were located above the linear regression line (see arrow heads in *Aretaon* panels in Fig. 7).

**Table 4:**
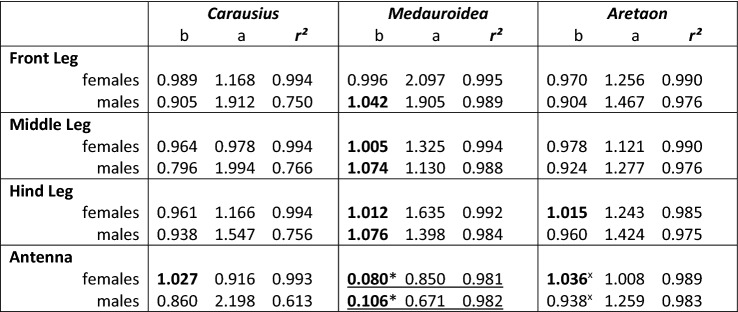
Allometry parameters

Scaling factor, *a*, and exponent, *b*, of allometric power functions as shown in Fig. 7. *r*^2^ gives the coefficient of determination of the log-transformed data, equivalent to the fraction of variance explained in log–log mappings. Exceptions are underscored parameters of *Medauroidea* antennae (curved functions in Fig. 7), where *b* is the scaling factor of the exponential exponent and *r*^2^ gives the variance explained in semi-logarithmic mappings. Bold numbers highlight positive allometric growth

*exponential fit; ^x^ power law fit to nymph data only

In all species investigated, the growth of the antennae markedly deviated from growth of the legs, though in different ways for each species. In *Carausius* females, antenna length grew according to an allometric power law, except that the exponent was considerably larger than those of the legs, resulting in a steeper slope of the linear regression in Fig. 7 (black line in top left panel). This was not the same in males, though the linear regression to the male sample explained 38% points less variance than the linear regression to the female sample (males: *r*^2^ = 0.613; females: *r*^2^ = 0.993). In *Medauroidea*, the growth of the antenna did not follow a power law but rather an exponential function. As a consequence, the log–log-transformed data have a curved distribution. The curvature can be explained very well by exponential fits, yielding coefficients of determination beyond 98% (Table 4). In *Aretaon*, growth of the antenna followed a power law for all nymphal stages, but then deviated strongly after the final moult. To illustrate this, linear regressions to antennal length were fitted for nymphal stages only (Fig. 7, black lines in *Aretaon* panels). Including adult antenna length resulted in larger allometry exponents (males: *b*_All_ = 1.148; *b*_Nymphs_ = 0.938; females: *b*_All_ = 1.105; *b*_Nymphs_ = 1.036), but the fits explained less variance than when fitted to the nymph data only (males: *r*^2^_All_ = 0.953; *r*^2^_Nymphs_ = 0.983; females: *r*^2^_All_ = 0.983; *r*^2^_Nymphs_ = 0.989). We conclude that antennal growth in *Aretaon* is best described by a power law for most of the postembryonic development, but underwent a boost during and/or after the last moult.

**Fig. 8:**
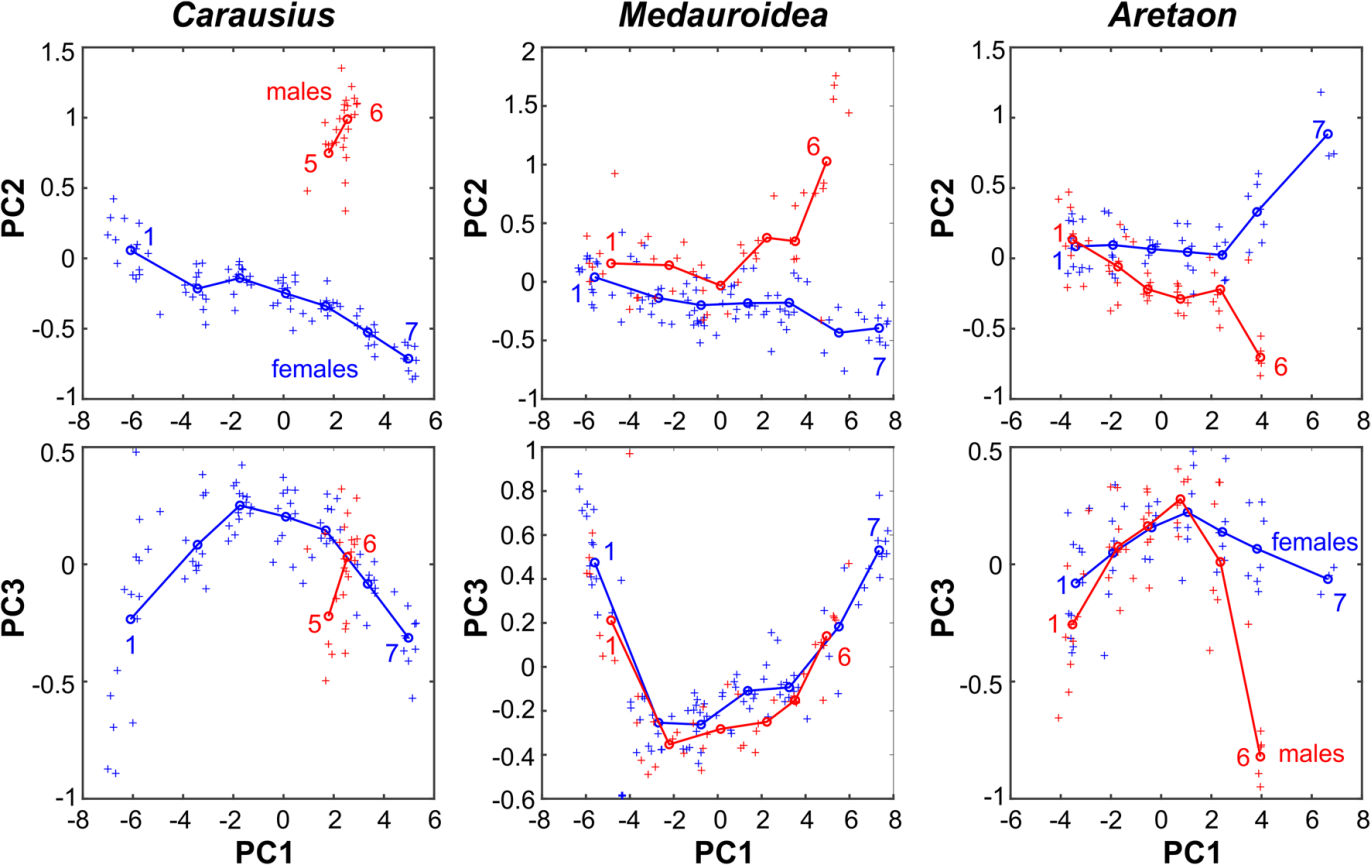
Sexual dimorphism develops most strongly from stage 5 onwards. Top panels plot scores of the *“general sexual dimorphism”* principal component 2 (PC2) against the *“growth”* principal component 1 (PC1). The latter explained more than 95% of the total variance in all species. Bottom panels plot the *“specific sexual dimorphism”* PC3 against PC1. Together, PC2 and PC3 clearly separate male from female specimens. Scattered symbols show scores of individual specimens (blue: females; red: males), circles and lines show mean scores per stage. Numbers next to the lines label the first and last instar per species and sex. Note that stage assignment was not part of the PCA and is used here for illustration only. Also note that this analysis includes only animals with all body measures labelled as “intact”, such that the original data set was slightly reduced to 111/115 in *Carausius*, 128/136 in *Medauroidea*, and 92/100 in *Aretaon*

Finally, we wanted to know how sexual dimorphism of limb proportions relates to sexual dimorphism of the overall body shape. To address this question, we took a multivariate morphospace approach that considered 45 linear body measures, including segment lengths of all limbs and length × width pairs for the head, thoracic and abdominal segments. Given the linearizing effect of logarithmic transformation (e.g., see Fig. 7), the resulting *N* × 45 measures per species were log-transformed and subject to Principal Component Analysis, PCA. PCA yields the eigenvectors of the covariance matrix with corresponding eigenvalues being proportional to the fraction of total variance explained. In our case, the first three principal components, PC1 to PC3, explained more than 97.9% of the total variance in the data sets, with PC1 already explaining at least 95.5%. The latter was because PC1 coded for the effect of “growth”, such that all coefficients of PC1 were positive and of little variation (mean ± s.d.: *Carausius* 0.148 ± 0.020; *Medauroidea* 0.147 ± 0.025; *Aretaon* 0.148 ± 0.017). The continuous effect of growth was mirrored by the fact that mean scores per stage were nearly equidistant for PC1, as illustrated by the left-to-right progression of mean values in Fig. 8 and by the much stronger effect on overall size than on shape in Suppl. S1. The colours in Suppl. Fig. S1 highlight that PC 1 also codes for some difference in limb-to-thorax length ratios (e.g. in front legs and antennae of *Carausius* and *Aretaon*). PC 1 also codes for some other features (e.g., relative tarsus length in *Medauroidea*) though all of these effects are considerably weaker and less consistent across species than its effect on growth.

**Fig. 9:**
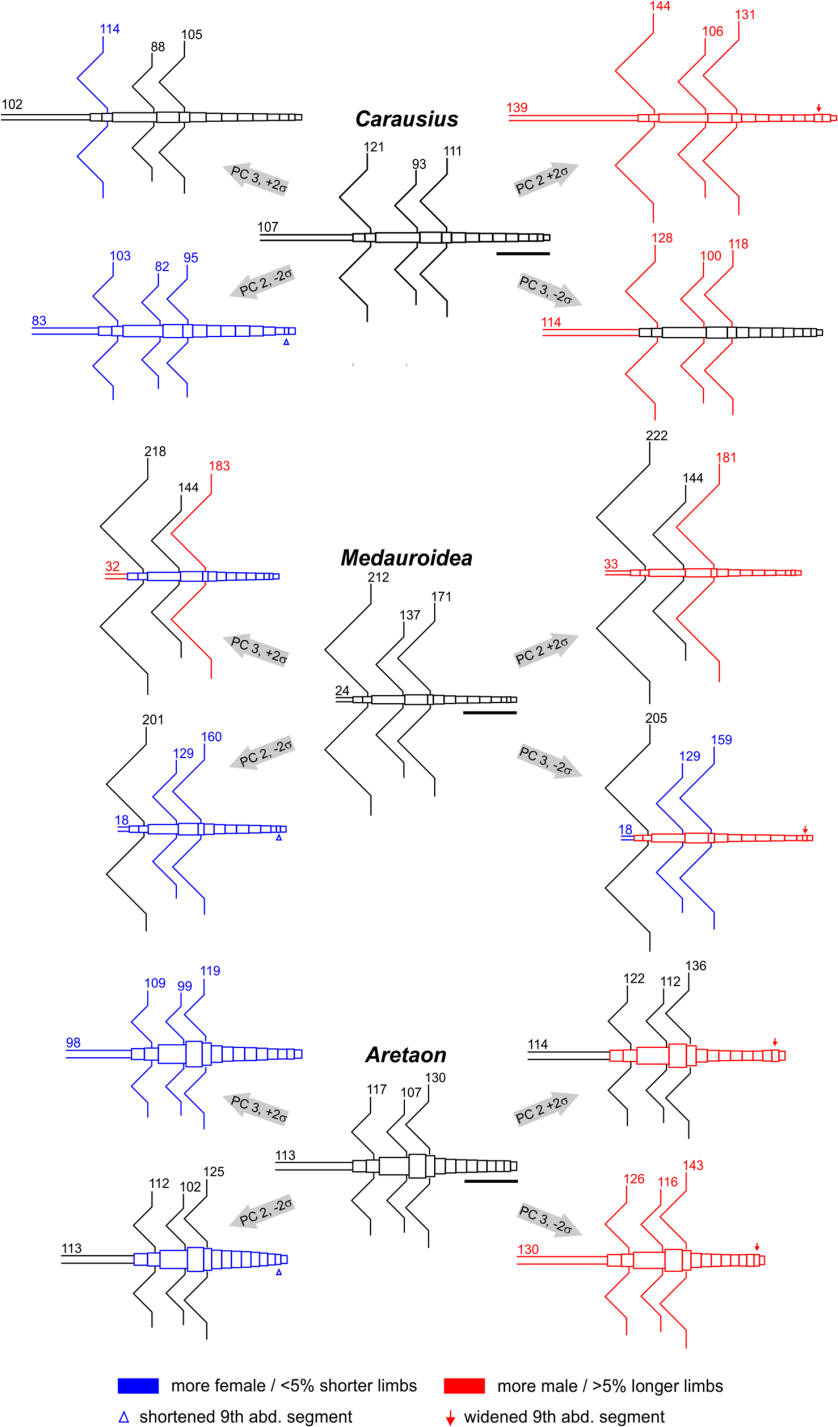
Principal components coding for sexual dimorphism, including changes in limb-to-thorax length ratios. Standardised mean body shapes (centres of each panel, below genus name) and their modulation according to PC 2 and PC 3 of the morphospace analysis (see also Fig. 8 and Suppl. Fig. S1). Grey arrows indicate respective PC number and sign of modulation. Magnitude of modulation was set to ± 2σ of the corresponding PC scores, thus spanning much of the range of variation within the data set. Scale bars are 10 mm. PC 2 and PC 3 have negligible effect on overall size, but code for changes in sexual dimorphism. Colours indicate more male (red) or more female (blue) features, compared to the mean (black). In case of the limbs, this means at least 5% deviation of the mean limb–to-thorax length ratios. Numbers next to the limbs indicate the limb-to-thorax length ratio in percent (for example, the mean front leg length of *Carausius* is 121% of its thorax length). Colours of the main body shape were assigned according to the overall appearance. Red arrows or blue arrow heads indicate a widened or shortened 9th abdominal segment as typical indicators of more female or more male body shape, respectively

PC2 explained between 1.1% of total variance in *Medauroidea* and 2.5% in *Carausius*. It coded for much of the sexual dimorphism by separating sex-specific increases and decreases of body measures by positive and negative coefficients, respectively. Despite the fact that PCA was calculated separately for each species, the patterns of positive and negative coefficients of PC2 were very consistent among species, making it the PC of *“general sexual dimorphism”*. For example, it coded for long limb segments (coefficients of antenna, femur, tibia and tarsus length all positive) but also for long and slender meso- and metathorax (positive coefficients for length, negative coefficients for width). PC2 also coded for other sexually dimorphic features that differed between species (Fig. 9). For example, in *Carausius* it contrasted increased width of abdominal segments 8 and 9 (the segments that bear the sexual organs) against decreased widths of all other abdominal segments. Mean scores per stage of PC2 clearly separated the sexes in all three species, as illustrated by the vertical separation of blue (female) and red (male) lines and symbols in the top row of Fig. 8.

PC3 explained between 0.6% of the total variance in *Carausius* and 1.15% in *Aretaon*. Like PC2, PC3 coded for sexually dimorphic features, though less consistently across species. Additionally, it appeared to code for features that were similar in 1st instar nymphs and adults, and different from 3rd, 4th, and 5th instar nymphs. Accordingly, plotting the mean scores per stage of PC3 against those of PC1 yielded curved trajectories that clearly separated the sexes for older nymphs (e.g. adult, 6th stage *Aretaon* males from 6th stage females; right lower panel in Fig. 8) but also 1st instar nymphs and adults on the one hand from 3rd to 5th instar nymphs on the other.

In summary, we conclude that the *“general sexual dimorphism”* PC2 together with the *“specific sexual dimorphism”* PC3 clearly separate male from female specimens. In *Medauroidea* and *Aretaon*, the distance between the sexes was small but distinct soon after the first moult (stage 2). As yet, it increased most strongly between stage 5 and stage 6, i.e., with the imaginal moult of males. In *Carausius*, the lack of young instars did not allow to determine how gradual or sudden the appearance of sexual dimorphism developed. However, because both PC2 and PC3 contrasted limb segment lengths against measures of the main body, and because the change in sexual dimorphism was strongest after the male imaginal moult in both species with samples from all stages, we conclude that sexual dimorphism of body:limb proportions occurs late in postembryonic development.

## Discussion

Following up on our species comparison of step parameters (Theunissen et al. 2015) and considerations about multiple behavioural functions of the so-called “walking legs” (Dürr et al. 2018), the present study compares the relative contributions of front legs and antennae to near-range exploration in three species with different body:limb proportions. Whereas it is clear that the three species cover much of the variation of the antenna-to-leg length ratio across the Phasmatodea, it is not clear whether the variation of this ratio is continuous within this order. We showed that the angular movement range of the antennae correlates with the antenna-leg length ratio (Figs. 2, 3), and that exploration volumes of antennae and front legs are complementary such that together they cover most of the frontal hemisphere about the head, with species-characteristic differences in the extent of medial overlap (Fig. 4). The subsequent morphometric analysis showed that the pattern of limb-to-thorax proportions in adults is species-characteristic, though sexually dimorphic (Fig. 5, Table 3). Throughout postembryonic development, we found that the antennae grow at higher rates than front legs, with different time courses of antennal growth among species (Fig. 7, Table 4). Finally, we showed that sexual dimorphism may be discernible early on in development, but changes most strongly during and/or after the fifth moult (Fig. 8).

### The pattern of body:limb proportions

Since all three of the species investigated are wingless and do not have specialised jumping legs, they are obligatory walkers, i.e., their only mode of locomotion is walking. Moreover, their nocturnal lifestyle implies that their sense of touch is particularly important for near-range exploration. Whereas the antennae are known to be important for tactile localisation of objects in walking and climbing *Carausius* (Dürr et al. 2001; Schütz and Dürr 2011; Krause and Dürr 2012; Berendes and Dürr 2022) and *Aretaon* (Bläsing and Cruse 2004), they are too short for tactile exploration in *Medauroidea*. Instead, the high swing movements of front legs in this species (Theunissen et al. 2015), their dense spatial coverage of the frontal hemisphere during walking (Figs. 2, 4) and the similar spatial ranges in walking and searching (Figs. 3, 4) clearly show that the front legs of *Medauroidea* effectively explore the near-range space during locomotion.

The exploratory function of antennae and front legs suggests that functional considerations about body:limb proportions should not be restricted to the legs but include the antennae. Moreover, since absolute and relative length ratios may differ considerably, the pattern of all limb lengths should be related to a reference measure of overall body size, such as thorax length (Fig. 6). For example, a look at the leg lengths of *Carausius* and *Medauroidea* in Fig. 5 reveals that their relative leg lengths are markedly different whereas the pattern of absolute leg lengths is very similar. Including relative antenna length further underscores their different patterns of body:limb proportions.

Since the differences among species were much larger than differences between the two sexes of the same species (Fig. 5), the pattern of body:limb proportions is species-characteristic, despite significant sexual dimorphism. Our allometry analysis (Fig. 7) suggests that the pattern of body:limb proportions changed gradually throughout postembryonic development, mainly because the growth rate of the antennae was consistently larger than that of most legs (different slopes or curvatures of black and coloured lines in Fig. 7). In contrast to the antennae, leg length proportions stayed very similar throughout postembryonic development (nearly parallel coloured lines in Fig. 7). A special case was *Aretaon*, as the relative length of its hind legs changed more during the last moult compared to all previous moults. Since *Aretaon* females have a protruding ovipositor, we propose that the long hind legs of *Aretaon* females may be necessary for appropriate lifting and/or positioning of the abdomen during oviposition into the substrate. Like in other Phasmatodean species with protruding ovipositor (e.g., *Eurycantha*; Boisseau et al. 2020), the hind legs are the longest legs in *Aretaon*, and this is case for both sexes and throughout postembryonic development (Fig. 7). Still, hind leg length increased most strongly during the imaginal moult.

Our morphospace analysis (Figs. 8, 9) suggests that sexual dimorphism of body:limb proportions develops by a rather sudden increase of relative growth in male 5th moult (Fig. 8). The apparent discrepancy with the allometry analysis (Fig. 7), where constant growth rates seem to support a gradual change of body–limb proportions, may be resolved by the particular role of antennal growth. In both *Medauroidea* and *Aretaon,* antennal growth increased most strongly in the last two stages. In *Carausiu*s, the situation remains unclear, mainly because of lacking young male nymphs and the associated uncertainty in assigning stage 5, but also due to the possibility of having mixed “true” males with masculinized females with male phenotype (Pijnacker and Ferwerda 1980).

Generally, male Phasmatodea tend to have larger limb-to-thorax length ratios than females, for walking legs as well as antennae. Aretaon is an example where the ratios are very similar in both sexes (Table 3). The sexual dimorphism of limb:body proportions is reflected by the finding that PC 2 and PC 3 clearly separate males from females (Fig. 8) and code for increments/decrements of limb-to-thorax length ratios in each one of the three species (Fig. 9), albeit to different degree. The increased relative limb length of males is likely to be reflected in different relative walking speeds (see above), though experimental data on this is lacking so far. Moreover, since males occur only sporadically in *Carausius* (at least in laboratory colonies) and *Medauroidea* strains are being bred in either bisexual (as in our case) or parthenogenetic colonies (Sven Bradler, personal communication), the biological function of longer limbs in male Phasmatodea is unclear, with few exceptions (see Boisseau et al. 2020). Potentially, comparative field work on naturally occurring stick insect populations with either bisexual or parthenogenetic reproduction (e.g., see Mantovani and Scali 1991 and Demontis et al. 2010 on the European species *Bacillus rossius*) may help to resolve this issue.

### Antennal movement and active sensing

Since the size of the antennal sampling volume immediately depends on flagellum length, volume *V* should scale allometrically with thorax length, *x*, according to *V* ~ (*a*·*x*^*b*^)^3^, with a and b according to Table 4. Allometric growth of the antenna differed considerably among the three species tested. In *Carausius*, relative growth rate remained constant throughout postembryonic development, but exceeding that of the front legs. This was similar in *Aretaon*, except that here growth rate increased suddenly during the final moult. In *Medauroidea* the antennae grew exponentially, i.e., with continuously increasing growth rate throughout postembryonic development. We conclude that the antennal sampling volume should grow faster than that of the front legs in all species investigated, a hypothesis that could be tested in future studies.

Since the Phasmatodea are almost exclusively nocturnal, their visual resolution (where it is known) is fairly poor (Jander and Volk-Heinrichs 1970), and targeted limb movements have only ever been reported in response to tactile cues (Schütz and Dürr 2011) rather than to visual cues (as reported for Orthoptera; e.g., Niven et al. 2010), we exclude the possibility that any of the species tested here use vision for near-range exploration or foot placement. Assuming that active movement of the antennae during locomotion serves tactile exploration of the near-range space, one might expect that species with particularly short antennae cannot use them for tactile localisation of obstacles and, therefore, should not invest energy in active antennal movement. Our observations on *Medauroidea* clearly show that this is not the case. Short antennae are moved during walking in similar ways as long antennae, albeit traversing a much smaller volume. We conclude that the purpose of antennal movement cannot be tactile sensing alone. Since insect antennae are multimodal sense organs and antennal movement in insects has been shown to be related to olfaction (e.g., Rust et al. 1976; Lent and Kwon 2004; Nishiyama et al. 2007) but also to perception of self-motion (Sane et al. 2007), future studies will need to identify the function of antennal movement in stick insects with short antennae. Our finding that the front leg movement range of *Medauroidea* leaves a “frontal slit” immediately ahead of the antennal movement volume (Figs. 2, 3) may be related to olfactory sampling efficiency. On the other hand, it is known that stick insects use their antennae for graviception (Wendler 1965), and it is unclear to what extent active movement may be part of that sensory function (Staudacher et al. 2005).

Our species comparison showed that antennal length correlates with the dorso-ventral extent of the antennal movement volume (Figs. 2, 3, 4), and that the regions of bilateral overlap differ in size and location (Fig. 4). In *Medauroidea*, the frontal location of the bilateral overlap region, along with its large relative size (8.3% of the total bilateral volume) fits to the location of the “frontal slit” discussed above, further hinting at a potential olfactory function of antennal movement. In *Carausius* and *Aretaon*, where antennae are known to serve as active tactile sensors, bilateral overlap regions should reflect increased (bilateral) effort for tactile exploration. If this is so, the frontal overlap region in *Carausius* should reduce the chance of missing obstacles immediately in front of the animal. In contrast, the very same frontal region was not sampled by *Aretaon* antennae*.* Instead, the major bilateral overlap region was located dorsally, directly above the head, with a further small overlap region in front and below the head. To what extent this distinctly different tactile exploration behaviour is related to differences in habitat and/or locomotion behaviour remains to be tested. Our own observations on wild-caught *Carausius morosus* on Madeira island suggest that this species mainly inhabits dense thickets (e.g., bramble) where it climbs about in the foliage at night. In our laboratory cultures, *C. morosus* spends little or no time on the cage floor, whereas adult *Aretaon asperrimus* commonly rest on the cage floor where the females place their eggs into the substrate. Future studies will need to tell to what extent the differences in antennal sampling behaviour indeed reflect differences in substrate preference.

### Leg length and leg function

It is trivial to observe that leg length immediately affects leg kinematics, simply because of an increased working range. Given the same leg posture, the same angular movement per stride and the same stride frequency, an animal with twice the leg length can move its feet twice as far per stride and, therefore, move twice as fast. In relative terms, however, large and small animals with equal body:limb proportions will move by the same number of body lengths per unit time. Assuming allometric scaling of leg and thorax length with an exponent close to unity, relative speed should stay the same. Indeed, large and small ants of the species *Cataglyphis bicolor* show the same overall gait parameters and reach the same relative speed (Tross et al. 2021). Of course, this kind of scaling requires that the body posture remains the same, irrespective of size. In the three species investigated here, leg posture during walking is not the same, as the ratio of body clearance over leg length is considerably smaller in *Medauroidea* than in *Carausius* and *Aretaon* (Theunissen et al. 2015). As this difference in posture translates into a difference in “effective leg length”, i.e. the distance of the basal leg joint to the substrate (Pontzer 2005), which in turn correlates with cost of transport for virtually all walking animals (Pontzer 2007), it is not surprising that *Medauroidea* does not walk at twice the speed of *Carausius* (Theunissen et al. 2015), despite their leg length differing by a factor of two. Consistent with the difference in posture, Theunissen et al. found *Medauroidea* to differ from the other two species with regard to step length distribution and overall step cycle kinematics, including swing height and joint angle ranges.

Limb posture also affects attachment forces (Büscher et al. 2020) that are generated by two functionally distinct types of attachment pads on the tarsi (Labonte and Federle 2013) that differentially affect tangential and normal attachment forces of the whole animal (Büscher and Gorb 2019). Posture dependency of attachment forces should also differ between the species used here, owing to different surface structure of tarsal attachment pads (nubby in *Carausius* and *Aretaon,* smooth in *Medauroidea;* Beutel and Gorb 2008; Busshardt et al. 2012).

Apart from its effect on locomotion, relative leg length has eco-physiological implications, particularly with regard to body temperature. In homeothermic animals, the volume-to-surface ratio of the body (which decreases with increasing relative limb length) correlates with altitude or latitude. However, this is less clear in poikilothermic animals: in European stick insects, the correlation is rather weak and inconsistent (Shelomi and Zeuss 2017). However, insects with long legs can lift their body higher above the ground, thus increasing body clearance above very hot substrates. For example, the desert-dwelling ant genera *Cataglyphis* and *Ocymyrmex* have independently evolved particularly large leg-to-thorax length ratios, with a correlation of latitude and relative leg length in *Cataglyphis* (Sommer and Wehner 2012). A similar correlation of latitude and relative leg length has been found in desert-dwelling darkling beetles (Broza et al. 1983).

With their “size-grain hypothesis”, Kaspari and Weiser (1999) proposed that relative leg length becomes less advantageous as animals become very small, mainly because the structural complexity of the environment (rugosity; locomotion in interstices) should favour shorter limbs. They found support for their hypothesis in ants, including a consistently higher allometric exponent for the tibia compared to the femur in five of the six ant subfamilies investigated. However, the size-grain hypothesis fails to explain allometric scaling of limb length in several other insect orders (Teuscher et al. 2009).

Recently, Boisseau et al. 2020) showed that sexual dimorphism of relative leg length may be related to the mating system, with larger and stronger legs being advantageous in male fighting behaviour. In the stick insect genus *Eurycantha* they found that males of a solitary, canopy-dwelling species have relatively shorter hind legs than the males of two gregarious, crevice dwelling species that fight over female mating partners. Our finding that all of the species used here show very little sexual dimorphism of limb-to-thorax length ratios (5) is consistent with the fact that none of them show fighting behaviour between males.

Finally, leg length may also affect mechanical properties such as the resistance to buckling which, assuming an allometric dependency of mass and leg length, will depend on the ratio of segment length over segment diameter (Prange 1977). Indeed, this ratio changes during postembryonic development in *Carausius*, though with corresponding changes in cuticle properties, thus maintaining resilience against buckling despite the more slender leg segment (Schmitt et al. 2018).

The last four examples clearly demonstrate that relative leg length not only affects terrestrial locomotion but has a much broader effect on the biology of a particular species. Our study adds a behavioural function that somewhat links locomotor ability with active-sensing ability: near-range exploration. The use of front legs in near-range exploration is supported by (i) the very large movement range of the front feet in *Medauroidea*, including regions remote from the substrate, and (ii) the complementary movement volumes of antennae and front legs in all species investigated, including (iii) an anti-correlation of front leg length and antennal length.

With regard to motor control, the relatively moderate differences in front leg exploration volumes and their bilateral overlap are consistent with the view that so-called searching movements of the front legs are equivalent to the swing movement phase of a step cycle (Dürr 2001; Dürr et al. 2018) until ground contact and associated load signals lead to a change in control mode (Dürr et al. 2018).

## Conclusions

Our study underscores the significance of front legs in near-range exploration and the complementary use of antennae and front legs. As in other studies on the relationship between leg length and leg function, this conclusion is based on correlation rather than on a causal relationship. As a consequence, it remains unresolved whether *Medauroidea* uses its front legs for near-range exploration because their length renders them suitable to do so, or whether they evolved long legs because the use of long legs in near-range exploration increased their fitness. Essentially, this is the same problem as in *Cataglyphis* ants, where it is not clear whether long-legged animals colonised increasingly hot areas or rather long legs evolved due to increased fitness in hot environments. Nevertheless, our conclusion leaves us to derive two testable hypotheses fur future studies.

First, the lengths of front legs and antennae should be anti-correlated across flightless, “obligatory walking” Phasmatodea, thus maintaining complementary near-range exploration volumes. We further predict that this relationship may be different in winged, actively flying species of Phasmatodea.

Second, we expect that the evolution of particularly long front legs gave rise to a "coupling of sensory and motor capabilities" in a way that is impossible for long antennae. An advantage for long front legs over long antennae could arise if tactile localisation of an object would immediately lead to firm grip and subsequent support during climbing. Thus coupling a tactile sensory ability with a locomotor ability in one and the same limb could be more efficient than "having the sensory antenna tell a front leg” where to reach. Accordingly, we predict that stick insects with increasingly longer front legs move about in habitats with increasing spatial complexity. In contrast, we would expect that particularly long antennae occurred in species that move in spatially less complex habitats, with extended surfaces.

## Supplementary Information

Below is the link to the electronic supplementary material.

Supplementary file1 (PDF 288 KB)
