# Supplementary material for: Behavioural function and development of body-to-limb proportions and active movement ranges in three stick insect species
Source: J Comp Physiol A Neuroethol Sens Neural Behav Physiol. 2022 Aug 20;209(2):265–84. doi: 10.1007/s00359-022-01564-z (PMC10006035; doi:10.1007/s00359-022-01564-z)
Supplement: Supplementary file 1 — Supplementary file1 (PDF 288 KB) [file 359_2022_1564_MOESM1_ESM.pdf]

Supplementary Material

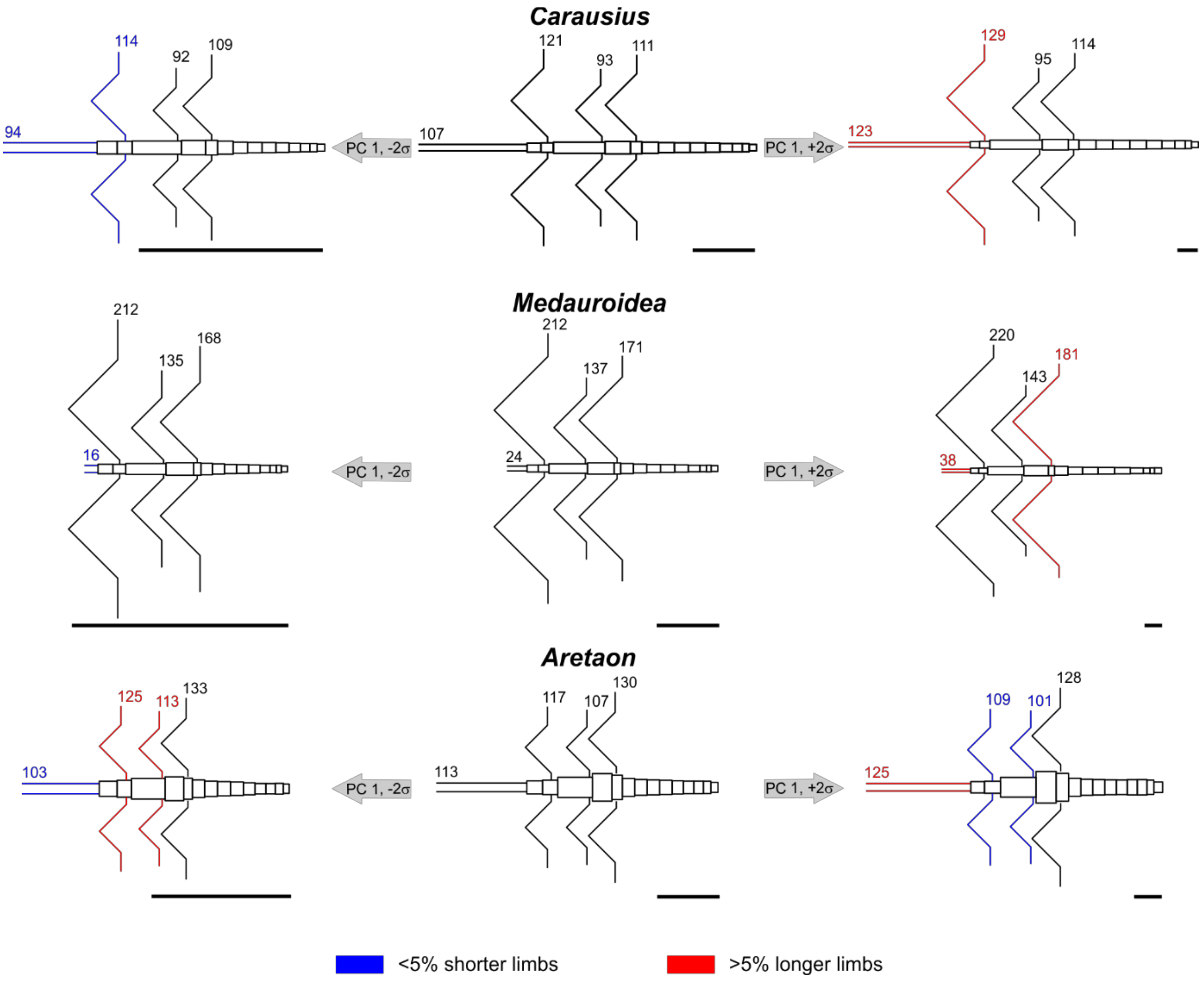

Supplementary Figure S1: Principal component coding for growth. Standardised mean body shapes (centres of each panel, below genus name) and their modulation according to PC 1 of morphospace analysis (see also Fig. 8 and Fig. 9). Grey arrows indicate the sign of modulation along the axis of PC 1. Magnitude of modulation was set to ±2σ of the corresponding PC scores to illustrate the range of variation within the data set. Scale bars are 10 mm, underscoring the strong effect of PC 1 on overall size. Additionally to its coding for growth, PC 1 also codes for some differences in overall body shape, including aspects of sexual dimorphism, though inconsistently. Colours of limbs indicate at least 5% larger (red) or 5% smaller (blue) limb-to-thorax length ratio compared to the mean (black). Numbers next to the limbs indicate the limb-length-to-thorax ratio in percent (for example, the mean front leg of *Carausius* is 121% longer than its thorax).

Dürr and Mesanovic, 2022: Behavioural function and development of body-to-limb proportions and active movement ranges in three stick insect species. Supplementary Material.

Supplementary Table S2: Principal Components of the Morphospace Analysis.

Top rows indicate the variance and cumulative variance explained by the first three PC of the morphospace analysis. Below that, columns list the 45 coefficients of each PC. Note that all coefficients of PC 1 are positive in all species, mirroring its effect on overall growth. PC 2 and PC 3 have both positive and negative coefficients, thus differentially increasing/decreasing the linear size of features of the antennae (Ant.) front legs (FL.), middle legs (ML.), hind legs (HL.), head (Hd.), thorax (T1. to T3.) or abdominal segments (A1 to A10.). Features include the position (.pos) of a limb base, the lengths of leg coxa (.cox), femur (.fem), tibia (.tib), tarsus (.tar), or the length (.L) or width (.W) of the head, thorax or abdominal segments.

	Carausius			***Medauroidea***			***Aretaon***		
	**PC1**	**PC2**	**PC3**	**PC1**	**PC2**	**PC3***	**PC1**	**PC2***	**PC3**
**variance**	95.52	2.5	0.6	96.34	1.1	0.76	95.52	1.28	1.15
**cumul. var.**	**95.520**	**98.020**	**98.620**	**96.340**	**97.440**	**98.200**	**95.520**	**96.800**	**97.950**
**Ant.pos**	0.098	-0.093	-0.117	0.104	-0.064	-0.172	0.115	-0.005	-0.006
**Ant.L**	0.176	0.292	-0.066	0.203	0.459	-0.376	0.171	0.094	-0.263
**FL.pos**	0.137	-0.070	0.019	0.123	-0.060	-0.095	0.184	0.170	0.299
**FL.cox**	0.150	-0.054	-0.046	0.168	-0.010	0.128	0.136	0.018	-0.085
**FL.fem**	0.165	0.201	-0.042	0.152	0.151	-0.005	0.137	0.036	-0.126
**FL.tib**	0.180	0.280	-0.116	0.166	0.170	-0.045	0.149	0.025	-0.218
**FL.tar**	0.137	0.153	-0.041	0.087	0.152	-0.067	0.141	0.052	-0.111
**ML.pos**	0.126	-0.119	0.244	0.137	-0.059	0.289	0.141	-0.004	-0.086
**ML.cox**	0.144	0.017	-0.116	0.191	0.037	0.007	0.143	0.005	-0.147
**ML.fem**	0.165	0.182	-0.059	0.153	0.150	-0.034	0.143	0.058	-0.147
**ML.tib**	0.167	0.217	-0.121	0.164	0.192	-0.094	0.145	0.009	-0.212
**ML.tar**	0.132	0.119	-0.036	0.100	0.136	-0.071	0.143	0.036	-0.125
**HL.pos**	0.148	-0.002	0.094	0.141	0.006	0.058	0.150	0.064	-0.061
**HL.cox**	0.149	-0.003	-0.014	0.183	0.052	-0.017	0.145	0.020	-0.137
**HL.fem**	0.167	0.213	-0.071	0.159	0.168	-0.061	0.150	0.054	-0.165
**HL.tib**	0.170	0.254	-0.102	0.166	0.179	-0.111	0.156	0.020	-0.231
**HL.tar**	0.126	0.127	-0.008	0.088	0.144	-0.091	0.140	0.043	-0.127
**Hd.L**	0.103	-0.094	-0.042	0.120	0.008	-0.134	0.120	-0.012	-0.058
**Hd.W**	0.105	-0.102	-0.078	0.117	-0.073	-0.152	0.118	-0.045	-0.046
**T1.L**	0.130	-0.047	-0.006	0.132	-0.038	-0.045	0.153	0.081	0.112
**T1.W**	0.112	-0.135	-0.068	0.121	-0.100	-0.120	0.114	-0.090	-0.023
**T2.L**	0.162	0.090	0.016	0.150	0.109	0.004	0.149	0.116	-0.109
**T2.W**	0.131	-0.152	-0.088	0.137	-0.166	-0.027	0.139	-0.060	0.114
**T3.L**	0.164	0.082	0.064	0.150	0.156	0.037	0.151	0.062	-0.055
**T3.W**	0.135	-0.176	-0.061	0.144	-0.205	0.006	0.169	-0.018	0.126
**A1.L**	0.149	-0.004	0.119	0.146	-0.006	0.093	0.176	0.151	0.038
**A1.W**	0.129	-0.194	-0.072	0.142	-0.208	-0.036	0.169	-0.070	0.109
**A2.L**	0.164	-0.001	0.278	0.166	0.071	0.308	0.144	0.082	0.052
**A2.W**	0.133	-0.198	-0.105	0.140	-0.214	-0.108	0.145	-0.287	0.135
**A3.L**	0.169	-0.030	0.268	0.168	0.059	0.261	0.138	0.122	0.072
**A3.W**	0.143	-0.209	-0.147	0.135	-0.222	-0.140	0.148	-0.380	0.084
**A4.L**	0.170	-0.062	0.283	0.173	0.025	0.235	0.136	0.136	0.121

Dürr and Mesanovic, 2022: Behavioural function and development of body-to-limb proportions and active movement ranges in three stick insect species. Supplementary Material.

**A4.W**	0.145	-0.224	-0.177	0.140	-0.233	-0.128	0.150	-0.384	0.082
**A5.L**	0.170	-0.072	0.293	0.177	0.002	0.282	0.134	0.147	0.185
**A5.W**	0.146	-0.222	-0.213	0.141	-0.234	-0.107	0.152	-0.383	0.092
**A6.L**	0.170	-0.083	0.290	0.176	-0.028	0.275	0.137	0.173	0.213
**A6.W**	0.148	-0.197	-0.207	0.141	-0.226	-0.129	0.161	-0.335	0.057
**A7.L**	0.167	-0.114	0.278	0.173	-0.043	0.276	0.138	0.142	0.233
**A7.W**	0.150	-0.114	-0.200	0.139	-0.218	-0.172	0.178	-0.240	-0.077
**A8.L**	0.164	-0.056	0.133	0.158	0.015	0.080	0.148	0.157	0.180
**A8.W**	0.157	0.041	-0.219	0.136	-0.139	-0.162	0.179	-0.030	-0.167
**A9.L**	0.145	0.292	0.101	0.152	0.018	0.079	0.128	0.173	0.264
**A9.W**	0.155	0.033	-0.191	0.137	-0.107	-0.072	0.169	0.095	-0.121
**A10.L**	0.151	-0.051	0.127	0.165	0.039	-0.045	0.181	-0.027	0.318
**A10.W**	0.142	-0.132	-0.028	0.155	-0.164	0.009	0.154	0.096	-0.028
